# Online Versus Classroom Teaching: Impact on Teacher and Student Relationship Quality and Quality of Life

**DOI:** 10.3389/fpsyg.2022.828774

**Published:** 2022-02-17

**Authors:** Paula Vagos, Lénia Carvalhais

**Affiliations:** ^1^Instituto de Desenvolvimento Humano Portucalense, Universidade Portucalense Infante D. Henrique, Porto, Portugal; ^2^Faculdade de Psicologia e Ciências da Educação, Centro de Investigação em Neuropsicologia e Intervenção Cognitivo-Comportamental (CINEICC), Universidade de Coimbra, Coimbra, Portugal

**Keywords:** student-teacher relationship, adolescence, online teaching, classroom teaching, quality of life

## Abstract

The student-teacher relationship (STR) has been consistently associated to positive and generalized outcomes, though its quality seems to be questioned in online teaching, which in turn has had a negative impact on students and teachers’ wellbeing during school closures forced by the COVID-19 pandemic. The current work compared students and teachers’ perceptions of STR quality and quality of life after online and after classroom teaching, and if STR quality relates with perceived wellbeing across those teaching modalities. Participants were 47 teachers (61.7% female, Mage = 47.85) and 56 students (48.2% female, Mage = 13.13), who self-reported on the quality of STR and quality of life twice: after 3 months of online teaching and after 3 months of classroom teaching. Quality of life remained stable across teaching modalities. Teachers perceived no differences in teacher-student quality across both moments; students perceived higher conflict after classroom teaching. Closeness in STR associated with increased wellbeing and the reverse was true for conflict, though diverse domains of quality of life were implicated across timings and across teachers and students. These findings concur to online teaching being an impersonal experience for students, where conflict is lower due to the absence of social stimuli; alternatively, teachers may be urged to use the STR as a resource to sustain better positive outcomes even when teaching online, both for them and for their students.

## Introduction

One of the many societal costs of the COVID-19 pandemic was school closures and the consequent interchange between online and classroom teaching. Though a public health necessity, online teaching took a tool on teachers’ mental health, who showed increases in anxiety (Li et al., 2020), exhaustion and burnout (Sokal et al., 2020), and intention to leave the profession (Zamarro et al., 2021). The health of adolescents was also negatively impacted by the pandemic, with children and adolescents reporting lower health-related quality of life, and higher mental health problems and anxiety symptoms (Ravens-Sieberer et al., 2021), particularly in relation to online teaching (Hyseni Duraku and Hoxha, 2020; Petillion and McNeil, 2020).

Several challenges appeared associated with online teaching, one of which named by both teachers and students has to do with restricted interactions opportunities (Hebebci et al., 2020). Online student-teacher interactions are thought of as non-authentic and lacking the spontaneity that in-person teaching provides (Tichavsky et al., 2015; Niemi and Kousa, 2020), leading students to prefer in-person courses because they provide for higher and closer interactions opportunities with teachers (Diebel and Gow, 2009; Tichavsky et al., 2015). This emotional connectedness is proposed to be an essential feature of positive teacher-student relationships (STR), either in person (Spilt et al., 2011) or online (Lai and Xue, 2012).

A positive and high quality of STR has been conceptualized based on low conflict (i.e., problematic relationship process between student and teacher) and high closeness (i.e., positive affect and communication between teacher and student; Pianta and Steinberg, 1992). Based on that conceptualization, the STR has been proposed to develop through contributions of both teachers and students (e.g., beliefs, attitudes, and behaviors), and impact on a myriad of positive outcomes experienced by students and teachers (for reviews on the subject see Hamre and Pianta, 2006; Myers and Pianta, 2008). Though the characteristics of the STR change across teaching levels (e.g., primary to secondary school, with secondary school teachers reporting that school organization and professional norms on interactions with students limit the opportunities of emotionally engaging with students; Hargreaves, 2000), this relationship remains an important contributor to older students’ academic and inter and intrapersonal adjustment within schools (Myers and Pianta, 2008; Roorda et al., 2011).

It remains to be determined if teaching modality (i.e., online versus classroom) changes the way STR manifests, given that previous literature has focused on one or the other (e.g., Longobardi et al., 2016; Hebebci et al., 2020). When comparisons were made between online and classroom teaching, STR was based on a general perception of positive relationships (Tichavsky et al., 2015), but not considering its specific closeness and conflict dimensions. Concerning quality of life, previous works suggested an increase in mental health difficulties following online teaching (e.g., Petillion and McNeil, 2020; Sokal et al., 2020), but considered only adults and mental health indicators. In turn, given that online teaching changes, for instances, the social and environmental context of learning, other dimensions that make up ones’ quality of life should be considered. Quality of life refers to ones’ idiosyncratic perception on ones’ functioning in relation to personal goals and cultural expectations and is applicable to diverse domains of life, namely, physical health (e.g., sleep patterns, experience of pain, energy, or mobility), psychological wellbeing (e.g., presence/absence of negative/positive feelings), social relations (e.g., perceived social support), and outer environment (e.g., perception of safeness or availability of resources for transportation, leisure; The WHOQOL Group, 1998). The way online teaching compares to classroom teaching concerning ones’ physical health, social relations, and perception of environment quality, in addition to psychological wellbeing has not been addressed. Finally, the association between STR and inter and intra personal positive outcomes has been found for classroom teaching (e.g., Myers and Pianta, 2008) but has not been addressed in the case of online teaching. Addressing these issues is the focus of the current work.

## Article Types

The current work is a brief research report using a repeated measures design to compare the perceived quality of STR and quality of life following two teaching moments (i.e., online followed by classroom teaching), as perceived by independent samples of teachers and students. Given that online teaching encompasses overall less opportunities for interacting (Diebel and Gow, 2009; Hebebci et al., 2020), we expect both closeness and conflict to be lower after online teaching: closeness would be lower, given that its absence is particularly referred to as a downside to online teaching (Tichavsky et al., 2015; Niemi and Kousa, 2020); conflict would also be lower, given that it depends strongly on interpersonal dynamics (Drugli, 2013), which may be absent in online teaching. As for quality of life, it is expected to be lower after online teaching, following previous work that proposes online teaching to have negatively impacted students (Hyseni Duraku and Hoxha, 2020; Petillion and McNeil, 2020) and teachers (Li et al., 2020; Sokal et al., 2020). Finally, another aim of this work is to analyze the association between STR and quality of life, across teaching modalities. Based on previous works that associate STR with positive individual-related outcomes (Pianta et al., 2003), we expect higher quality of STR to associate with higher quality of life (and vice-versa) after classroom teaching; as evidence is scarcer on that association after online teaching, we expect to find the same direction of association.

## Materials and Methods

### Participants

#### Teacher Sample

Forty-seven teachers at public schools composed the teacher sample. They were aged between 28 and 61 years old (M = 47.85, SD = 6.54) and taught from the first to the fourth grade (*n* = 3), from the fifth to the ninth grade (*n* = 39), and from the 10th to the 12th grade (*n* = 5). Concerning gender, 61.7% (*n* = 29) of these teachers were female and 38.3% (*n* = 18) were male. Most teachers were married/co-habited with a significant other (*n* = 40, 85.1%) and were full-time employed (*n* = 44, 93.6%). Female and male participants had similar mean ages [*t*_(4)_ = −0.29, *p* = 0.77] and were similarly distributed concerning marital status [*χ*^2^_(2)_ = 2.14, *p* = 0.34] and professional situation [*χ*^2^_(1)_ = 1.98, *p* = 0.16].

#### Student Sample

Fifty-six students attending urban schools and aged between 12 and 15 years old (*M* = 13.13, SD = 0.92) comprised our student sample, of which 29 were male (51.8%) and 27 were female (48.2%). They attended the seventh (*n* = 31. 55.4%), the eighth (*n* = 15, 26.8%), and the ninth (*n* = 10, 17.9%) grades; only a minority of them had been previously retained in the same school year (*n* = 2, 3.6%). Boys and girls were similarly distributed by school year [*χ*^2^_(2)_ = 0.29, *p* = 0.87] and had similar mean ages [*t*_(54)_ = 0.99, *p* = 0.33].

### Instruments

All instruments were used in their Portuguese version.

#### Teacher Protocol

##### Short Form of the Student-Teacher Relationship Scale (STRS-SF)

This self-report instrument includes 15 items according to which teachers report on their perception of student-teacher relationship, conceptualized as low levels of conflict and high levels of closeness (Pianta, 2001). Results on its Portuguese version using a 5-point scale ranging from “Definitely does not apply” to “Definitely applies” presented with very good psychometric properties, namely: good internal consistency (*α* < 0.86), internal structure validity based on two measures *via* confirmatory and exploratory factor analyses, and sensitivity to diversity by sex (Patrício et al., 2015). Its results using the current sample obtained very good internal consistency values for closeness (*α* = 0.70 after-online teaching and *α* = 0.78 after-classroom teaching) and conflict (*α* = 0.83 after-online teaching and *α* = 0.84 after-classroom teaching).

##### WHOQOL Bref (Bref)

The WHOQOL Bref is a self-report instrument composed of 26 items intended to address quality of life referring to several domains relevant to adults: physical wellbeing, psychological wellbeing, social relationships, and environment (The WHOQOL Group, 1998). Results on its Portuguese version presented with at least acceptable internal consistency for each of the four domains (*α* > 0.64), temporal stability, and construct validity in relation to measures of psychopathology and depression (Canavarro et al., 2010). Using the current sample, internal consistency values for scores on all domains were very good: *α* = 0.84 after-online teaching and *α* = 0.86 after-classroom teaching for physical wellbeing, *α* = 0.87 after-online teaching and *α* = 0.80 after-classroom teaching for psychological wellbeing, *α* = 0.86 after-online teaching and *α* = 0.75 after-classroom teaching for social relationships, and *α* = 0.77 after-online teaching and *α* = 0.76 after-classroom teaching for environment.

#### Student Protocol

##### Short Form of the Student-Teacher Relationship Scale—Student Version (STRS-Student)

This self-report instrument resulted from the adaption of the STRS-SF (see Maia et al., 2020) to be filled in by students and assess the same two dimensions proposed to be part of the quality of STR (i.e., closeness and conflict; Pianta, 2001). It includes 16 items (i.e., item 10 from the STRS-SF was split into two items that differentiate the option of becoming angry when being disciplined by teachers and of not complying with orders received during that disciplining) that the student uses to characterize his/her relationships with teachers in general, using a 7-point scale ranging from “Has nothing to do with me” to “Has everything to do with me.” Its two-factor internal structure was confirmed using a Portuguese adolescent sample and both factors showed good internal consistency values and invariance by gender (Maia et al., 2020). At least good internal consistency values were found for the measures of this instrument within the current sample: *α* = 0.81 for closeness at both assessment moments, and *α* = 0.80 and *α* = 0.83 for conflict, after-online and after-classroom teaching, respectively.

##### KIDSCREEN 27

The KIDSCREEN 27 (Ravens-Sieberer et al., 2014) was designed to assess children’s and adolescents’ perception of wellbeing and health. Its 27-item version, which was used in the current work, assesses physical wellbeing, psychological wellbeing, autonomy and parent relations, peers and social support, and school environment. The Portuguese version of this instrument is said to be sensitive to differences based on gender, age, socioeconomic status, and health condition, with findings being overall similar to its full 52-item version (Gaspar and Matos, 2008). Internal consistency values for domains were good using the current sample: *α* = 0.77 after-online teaching and *α* = 0.80 after-classroom teaching for physical wellbeing; *α* = 0.91 after-online teaching and *α* = 0.89 after-classroom teaching for psychological wellbeing; *α* = 0.73 after-online teaching and *α* = 0.78 after-classroom teaching for autonomy and parent relation; *α* = 0.74 after-online teaching and *α* = 0.83 after-classroom teaching for peers and social support; and *α* = 0.77 after-online teaching and *α* = 0.80 after-classroom teaching for school environment.

### Procedures

This study was conducted after approval by the National Education Ministry (Inquiry number 0617900005) and the Ethics Committee at the host institution. The student sample was recruited in school settings. Two schools located at the center region of Portugal were asked to take part of this research and sent informed consents to the parents/legal guardians of students attending the seventh through ninth grades. Students with parental consent were then asked verbal assent to fill in the research protocol in class using time made available by teachers. The teacher sample was collected online using the google forms platform, where teachers filled in an informed consent form before replying to the research protocol. The study was divulged *via* social media platforms and each participant was asked to refer other potential participants to the study.

Data collection took place during one single school year. The first assessment moment occurred in the first week of April 2021, when students and teachers had just came back to schools after a confinement period of 3 months in Portugal (from mid-January to March 2021); this corresponds to the after-online teaching moment. The online experience consisted of classes being held *via* synchronized videoconferences (i.e., Google Teams), when teachers were expected to be present throughout the sessions and to abide by the expected teaching-learning agenda for each course. Then, the same students and teachers were asked to fill in the same research protocols at the end of June, after having been in classroom teaching for about 3 months; this corresponds to the after-classroom teaching moment. A *priori* power analyses indicated that a sample size of at least 47 participants would be needed to find medium effect size differences between two matched samples using non-parametric analyses, with type 1 error fixed at *p* = 0.05. Given that most of our measures did not follow a normal distribution, non-parametric statistics were used to analyze the data.

## Results

### Teachers’ Perception of Teacher-Student Relationship and Its Associations With Quality of Life

No significant differences were found for teachers’ perception of the quality of the relationship with their students, either for closeness (*z* = −0.12, *p* = 0.91, *r* = −0.01) or for conflict (*z* = −1.14, *p* = 0.25, *r* = −0.12). Similarly, no significant differences were found across time for physical wellbeing (*z* = −0.37, *p* = 0.71, *r* = −0.04), psychological wellbeing (*z* = −0.03, *p* = 0.98, *r* = −0.00), social relationships (*z* = −0.11, *p* = 0.91, *r* = −0.01), or environment (*z* = −0.56, *p* = 0.57, *r* = −0.06). See Table 1 for descriptive values for both moments.

**Table 1:** Descriptive values for teacher-student relationship and wellbeing, for teachers and for students.

	After online teaching	After classroom teaching
**Teacher sample**		
Closeness	28.08 (3.63)	28.06 (3.67)
Conflict	17.98 (5.81)	18.85 (6.16)
Physical wellbeing	27.43 (4.95)	27.45 (4.58)
Psychological wellbeing	23.81 (4.25)	24.00 (3.27)
Social relationships	11.64 (2.82)	11.62 (2.41)
Environment	30.51 (9.98)	30.72 (4.09)
**Student sample**		
Closeness	19.83 (5.67)	19.29 (5.71)
Conflict	16.96 (5.48)	18.78 (6.51)
Physical wellbeing	16.14 (2.67)	16.00 (2.90)
Psychological wellbeing	27.67 (5.46)	27.09 (5.49)
Autonomy and parent relation	25.56 (3.48)	25.30 (4.00)
Peers and social support	17.02 (2.649)	17.42 (82.68)
School environment	15.32 (2.87)	14.71 (3.46)

*Data are presented as M (SD)*.

After-online teaching, closeness associated positively and significantly with social relationships (*r_s_* = 0.23, *p* = 0.04). After-classroom teaching, conflict associated negatively and significantly with psychological wellbeing (*r_s_* = −0.29, *p* = 0.04) and with environment (*r_s_* = −0.352, *p* = 0.02). No other significant correlation values were found between STR and quality of life using the teacher sample.

### Students’ Perception of Teacher-Student Relationship and Its Associations With Quality of Life

A significant difference was found for conflict (*z* = −3.03, *p* = 0.002, *r* = −0.29), with higher scores being found after classroom teaching in comparison with after online teaching; the same comparison for closeness was not statistically significant (*z* = −1.14, *p* = 0.26, *r* = −0.11). No significant differences were found across teaching modalities for physical wellbeing (*z* = −0.65, *p* = 0.52, *r* = −0.06), psychological wellbeing (*z* = −1.58, *p* = 0.12, *r* = −0.14), autonomy and parent relation (*z* = −0.42, *p* = 0.68, *r* = −0.04), peers and social support (*z* = 0.90, *p* = 0.37, *r* = −0.01), and school environment (*z* = −1.93, *p* = 0.05, *r* = −0.18; see Table 1).

Consistently across teaching modalities, closeness associated positively and significantly with psychological wellbeing (*r_s_* = 0.28, *p* = 0.04 after-online teaching and *r_s_* = 0.29, *p* = 0.03 after-classroom teaching) and school environment (*r_s_* = 0.45, *p* = 0.001 after-online teaching and *r_s_* = 0.35, *p* = 0.008 after-classroom teaching). Also consistently, conflict correlated significantly and negatively with school environment after-classroom teaching (*r_s_* = −0.49, *p* < 0.001) and after-online teaching (*r_s_* = −0.50, *p* < 0.001). After-online teaching only, conflict also correlated significantly and negatively with psychological wellbeing (*r_s_* = −0.35, *p* = 0.01), whereas after-classroom teaching only it correlated significantly with physical wellbeing (*r_s_* = −38, *p* = 0.005) and autonomy and parent relation (*r_s_* = −0.36, *p* = 0.007) dimensions. No other significant correlations values were found between STR and quality of life using the student sample.

## Discussion

This work set out to compare perceived quality of STR, as reported by teachers and students, after-online and after-classroom teaching. Previous findings highlight the lack of interaction opportunities as a downside to online teaching (e.g., Tichavsky et al., 2015; Niemi and Kousa, 2020) but have not address how that compares to classroom teaching for adolescents, who more strongly crave for social contacts (Orben et al., 2020), nor based on an operationalized and comparable conceptualization of that relationships based on closeness and conflict. Also, we wanted to verify the associations between STR and quality of life after both teaching modalities, following previous findings that online teaching impacted on the mental health of both teachers and students (Hyseni Duraku and Hoxha, 2020).

Findings only partially confirm our hypotheses concerning conflict and closeness after-online teaching compared to after-classroom teaching: the difference was only statistically significant for students’ perception of conflict. The conflict dimension of the STR has been associated specifically to the behaviors that students practice in school contexts: STR has been found to both predict (Drugli, 2013; Longobardi et al., 2016) and be predicted (Rudasill et al., 2010; Longobardi et al., 2018) by those behaviors. Besides interaction opportunities likely being lower in online teaching (Diebel and Gow, 2009), thus resulting in less opportunities for conflict, this effect may be particularly pronounced for adolescents who lack the self-regulatory skills that have been proposed to facilitate involvement in online teaching (Tichavsky et al., 2015; Flores et al., 2021).

As for the associations between STR and quality of life reported by students, they were more consistent for closeness than for conflict. Positive associations were consistently found across teaching modalities between closeness and psychological wellbeing and school environment, mirroring what was previously found as outcomes for positive STR relationships (Bernstein-Yamashiro and Noam, 2013). Another consistent association was found between conflict and a negative perception of the school environment; students who are in conflict with teachers may seek the comfort/approval from peers who face similar experiences (Rudasill et al., 2010) and, inadvertently, that may lead them to be further alienated from both teachers and peers, thus leading to a negative perception of the school environment. No significant differences were found for quality of life across teaching modalities, unlike previous findings (e.g., Petillion and McNeil, 2020); the fact that we explored several domains of quality of life, instead of specific symptoms (e.g., motivation, stress, or anxiety) may have sustained these diverse findings. It nevertheless seems worth mentioning the near-significant difference found for school environment, which had a lower score after classroom teaching. This may be another way of students expressing a more generalized conflict-based interaction pattern of relating to both teachers and peers. As for the way STR associated with conflict, it only negatively associated with psychological wellbeing after online teaching; the fact that this was not found after-classroom teaching may have to do with the biggest emphasis on social aspects of wellbeing when opportunities for in-person interactions are available. Conflict after-classroom teaching associated with physical wellbeing and autonomy and parent relations. On the one hand, and because conflict with teachers usually also reflects in conflict with peers (Longobardi et al., 2018; Maia and Vagos, 2021), schools may be a context where face-to-face overt aggression acts are possible (because students are in the same physical place), which impacts on physical wellbeing. On the other hand, after having lived in close quarters with parents and away from teachers, adolescents may be experiencing a renewal in their developmental task of negotiating their autonomy from adults (Moretti and Peled, 2004), which may be reflected both in conflict with teachers and perceived difficulties in managing parental relationships.

Teachers’ perception of STR was unchanged when comparing after-online with after-classroom teaching. Teachers have been found to be more focused on the technical difficulties of teaching online (e.g., available resources and infrastructures; Trust and Whalen, 2020). This, plus the fact that the sample is composed mostly of teachers of older students who consider emotional connections and expressions to be less relevant (Hargreaves, 2000), may have made them less aware of changes to the way they relate with their students. After online teaching, teachers’ perception of closeness associated with their satisfaction with social relationships. Feeling closer to ones’ students is proposed to be another way of satisfying teachers’ needs for connection (Spilt et al., 2011) and has actually been found to fulfils this role, at least for some teachers (O’Connor, 2008). This, in addition to the fact that while confined teachers may have had more opportunities to connect with significant others (e.g., family members), may have made this period when online teaching occurred particularly satisfying concerning the social domains of teachers’ life. After-classroom teaching, teachers’ perception of conflict associated with diminished psychological wellbeing and with a negative perception of their surroundings. Classroom teaching may demand more of teachers, namely, in the way they connect with students, manage students’ and their owns’ behavior, and are subjected to students’ potentially reactive and quarrelsome interaction patterns (O’Connor, 2008). These demands may negatively impact on their psychological wellbeing and the way they perceive their (working) environment.

### Implications for Applied Settings

Because STR are available to all students (Myers and Pianta, 2008), its quality may be improved so that STR may be a resource put to use to contribute to better personal and professional/academic outcomes, in both students and teachers (Pianta et al., 2003). Interventions aimed at promoting higher quality in STR have growingly received attention and empirical support (Hamre and Pianta, 2006), namely, by helping teachers recognize the relevance of classrooms as developmental and communication contexts and actively using them as such (Pianta et al., 2012). This line of thinking should also be applied to online teaching. Online education has been thought of as encompassing a lack of emotion in STR, where the focus is on knowledge reporting without any emotional communication being involved in the learning process (Lai and Xue, 2012). Alternatively, helping teachers and students develop not only the technical but also the socio-emotional competencies to manage online learning and secure a significant online social and supportive presence, may result in a better overall online teaching experience (Flores et al., 2021).

### Limitations

Though teaching modality was clearly differentiated in the current work (i.e., online implied online contact only whereas classroom implied in-person contact only) and lasted for the same amount of time, we could not distinguish the effects of teaching modality from potential longitudinal (i.e., derived from the passing of time across the school year, which in this case corresponded to transitioning from online to classroom teaching) or learning effects (i.e., producing similar responses from one moment to another based on recollection of what was answered in the first assessment moment). Having had access to other groups who had gone through the opposite transition (i.e., from classroom to online teaching) or using independent (but homogenous) groups who experienced different teaching modalities at the same time would be an important way to further compare and explore current findings in the future. Nevertheless, because previous literature indicates conflict to be stable but closeness to be less so (Roorda et al., 2011) and we did not replicate those outcomes, particularly for students, we may expect that teaching modality was the main precursor of current findings. Also, we did not assess dyads so the reciprocal effects of closeness and conflict among teachers and students could not be inferred, but only proposed and discussed based on existing literature (e.g., Baker et al., 2008; Longobardi et al., 2016). Future works considering the mutual perceptions of student-teacher dyads would allow a closer look into the characteristics and evolution of student-teacher relationships across diverse demands, such as online versus classroom teaching. Finally, our sample size and sites where data were collected are limited, which implies caution when generalizing current findings and prevented us from analyzing other person-related variables that may have impacted current findings, such as, to name only a few, student or teacher gender (Drugli, 2013) or resources available to implement diverse online- and classroom-based teaching strategies (Hebebci et al., 2020).

### Conclusion

Online teaching, as practiced during one of the confinement periods in Portugal, seemed to be an impersonal experience, where neither closeness nor conflict arose in comparison with classroom teaching. Though lower conflict may be thought of as positive, it does not seem to derive from increased social abilities nor the establishment of positive interactions, but rather from the absence of interpersonal stimuli. So, following previous assumptions (Orben et al., 2020), another consequence of social isolation may be the underdevelopment of social competences needed for students’ interpersonal adjustment in the long run. Alternatively, neither online nor classroom teaching was superior in relation to providing feelings of closeness and connectedness between teachers and students but that does not have to be the case. As educational practices evolve to accommodate online teaching and other technology mediated teaching strategies as prompted by the COVID-19 pandemic (Poletti, 2020; Stoller, 2021), teachers should be encouraged to establish a social presence and connect with students, thus contributing not only to their own quality of life but also to the whole development of their students.

## Data Availability Statement

The raw data supporting the conclusions of this article will be made available by the authors, upon reasonable request.

## Ethics Statement

The studies involving human participants were reviewed and approved by the Comissão de Ética para a Saúde da Universidade Portucalense Infante D. Henrique. Written informed consent to participate in this study was provided by the participants’ legal guardian/next of kin.

## Author Contributions

PV was responsible for the design of the study and for describing the methods and results sections of the manuscript. LC was responsible for the introduction and discussion section of the manuscript. PV and LC contributed to the validating each other’s responsibilities and to the writing of the manuscript in its current presentation. All authors contributed to the final version of the article and approved the submitted version.

## Funding

Universidade Portucalense Infante D. Henrique funded this work by supporting its publication fees but was not involved in the design of the study, analyses of its outcomes, or preparation of the manuscript.

## Conflict of Interest

The authors declare that the research was conducted in the absence of any commercial or financial relationships that could be construed as a potential conflict of interest.

## Publisher’s Note

All claims expressed in this article are solely those of the authors and do not necessarily represent those of their affiliated organizations, or those of the publisher, the editors and the reviewers. Any product that may be evaluated in this article, or claim that may be made by its manufacturer, is not guaranteed or endorsed by the publisher.
